# Validity and reproducibility of a whole‐room indirect calorimeter for the estimation of VO_2_ , VCO_2_ , and resting metabolic rate

**DOI:** 10.14814/phy2.15658

**Published:** 2023-04-05

**Authors:** Hege Berg Henriksen, Christine Henriksen, Ana Rita Sequeira de Sousa, Dena Treider Alavi, Elin Maria Sandstad Augestad, Russell Rising, Rebecca Dörner, Franziska Anna Hägele, Anja Bosy‐Westphal, Rune Blomhoff, Stine Marie Ulven, Thomas Olsen

**Affiliations:** ^1^ Department of Nutrition, Faculty of Medicine, Institute of Basic Medical Sciences University of Oslo Oslo Norway; ^2^ D & S Consulting Services, Inc. New York New York USA; ^3^ Institute of Human Nutrition and Food Science Christian‐Albrechts University Kiel Germany; ^4^ Division of Cancer Medicine, Department of Clinical Service Oslo University Hospital Oslo Norway

**Keywords:** energy expenditure, metabolism, resting metabolic rate, whole‐room indirect calorimetry

## Abstract

Whole‐room indirect calorimeters (WRICs) provide accurate instruments for the measurement of respiratory exchange, energy expenditure, and macronutrient oxidation. Here, we aimed to determine the validity and reproducibility of a 7500 L WRIC for the measurement of ventilation rates and resting metabolic rate (RMR). Technical validation was performed with propane combustion tests (*n* = 10) whereas biological reproducibility was tested in healthy subjects (13 women, 6 men, mean ± SD age 39.6 ± 15.3) in two 60 min measurements separated by 24 h. Subjects followed a run‐in protocol prior to measurements. The coefficient of variation (CV) and intraclass correlation coefficient (ICC) were calculated for ventilation rates of O2 (VO2), CO2 (VCO2), the respiratory quotient (RQ; VCO2/VO2), and RMR. Technical validation showed good validity with CVs ranging from 0.67% for VO_2_ to 1.00% for energy expenditure. For biological reproducibility, CVs were 2.89% for VO_2_; 2.67% for VCO_2_; 1.95% for RQ; and 2.68% for RMR. With the exception of RQ (74%), ICCs were excellent for VO_2_ (94%), VCO_2_ (96%) and RMR (95%). Excluding participants that deviated from the run‐in protocol did not alter results. In conclusion, the 7500 L WRIC is technically valid and reproducible for ventilation rates and RMR.

## Graphical abstract

Whole‐room indirect calorimetry is an accurate method for measuring metabolic rates. Here, we report that a newly built whole‐room indirect calorimeter at the Department of Nutrition, University of Oslo provides valid and reproducible measurements of resting metabolic rate.

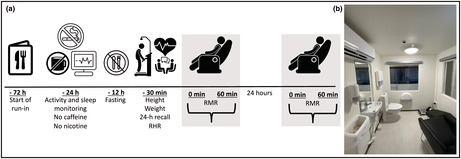

## 1 INTRODUCTION

Whole‐room indirect calorimeters (WRICs) provide accurate instruments for the measurement of respiratory exchange, energy expenditure, and macronutrient oxidation, and are currently in use at more than 40 research facilities globally (Chen, Smith, et al., 2020). Modern WRICs accurately capture components of total daily energy expenditure (TDEE) over a 24 h measurement period (Allerton et al., 2021; Dörner et al., 2022; Stinson et al., 2022). WRICs can be used to study energy requirements in humans with different metabolic conditions and to study the effects of different dietary exposures on the various components of energy expenditure (Hall et al., 2019, 2021; Müller et al., 2021).

Resting metabolic rate (RMR) makes up about 70%–85% of TDEE, whereas physical activity accounts for 15% to 30%, and is otherwise influenced by age, gender, hormones, body size, body composition, physical activity, dietary factors, and diseases (Bosy‐Westphal et al., 2009; Heymsfield et al., 2018, 2019, 2021; Ruggiero & Ferrucci, 2006). Therefore, accurate tools for the measurement of RMR are important in research and the clinic. Easy‐to‐use tools for the measurement of RMR include ventilated hoods that offer time‐ and cost‐effective alternatives to a 24‐h measurement in a WRIC, and predictive equations that are commonly used in clinical practice. Both methods are less accurate and imprecise compared to whole‐room indirect calorimetry (Rising et al., 2015). In this regard, smaller WRICs offer an attractive alternative as it has shorter response times compared to WRICs built for 24‐h measurements (Chen, Smith, et al., 2020). The short response time enables the detection of metabolic changes in a study subject or patient within 30 to 60 min with a high degree of accuracy (Rising et al., 2015, 2017).

At the Department of Nutrition, University of Oslo, Norway, a small WRIC with an interior volume of 7500 Ls has been built to facilitate measurements of RMR. According to newly established guidelines for using and reporting WRIC facilities, it is recommended that validation and reproducibility experiments on energy expenditure (EE) and metabolic rates are performed and published (Chen, Smith, et al., 2020). The aim of this study was therefore to validate a WRIC intended for measurements of RMR. Validation was performed using propane gas combustion tests (technical validation), and two 60 min measurements separated by 24 h in healthy men and women to ascertain the reproducibility/biological validity of VO_2_, VCO_2_, RMR, and the respiratory quotient (RQ).

## 2 MATERIALS AND METHODS

### 2.1 System characteristics, data acquisition, and processing

Three rooms have been built at the Department of Nutrition, University of Oslo, Norway for the measurement of metabolic rates over 24 h, during exercise and at rest. The present study measured and reports RMR (kcal/d) and the respiratory quotient (RQ; L (V) of CO_2_/L of O_2_) measured using the WRIC specific for RMR. This involved the determination of oxygen (O_2_, %), carbon dioxide (CO_2_, %), and water vapor pressure (WVP, kPa) according to previously published protocols (Rising et al., 2015, 2017). The RMR‐WRIC chamber has an interior volume of 7500 L after accounting for space taken up by a recliner, sink, and toilet. It has one window with a view of outside surroundings and one window facing the control room. The door is equipped with a blood sample port that was not in use for this particular study.

Fresh outside air is infused into the buffer spaces surrounding the WRIC by the main building's heating, ventilation, and air‐conditioning system. This system ensures a slight overpressure to prevent any outwards leaks from the WRIC and maintains an air temperature of 22°C. Air is directed from the buffer space into the WRIC at floor level. The WRIC is equipped with a heat pump unit (LG D09TR NSJ, LG Electronics) that maintains a temperature of 22°C and set at a low fan speed to mix respiratory gases. Air is continuously pulled from the RMR‐WRIC at a constant flow rate of 100 L/min, and O_2_, CO_2_, and WVP concentrations are measured by a Promethion GA3m2/FG‐250 (Sable Systems International) integrated metabolic instrumentation. This instrumentation contains dual CO_2_, O_2_, and WVP sensors that alternate between measuring gas concentrations in the incurrent (baseline air from the buffer space) and excurrent air streams, thus maintaining a continuous measurement of the subject's respiratory exchange within the RMR‐WRIC.

Raw data were acquired by Caloscreen 1.3.16 (Sable Systems International) and processed by ExpeData 1.9.27 (Sable Systems International). This software contains a macro code for mathematically processing the data including signal preconditioning, WVP dilution correction, and conversion to standard pressure, as well as baseline drift correction from incurrent air measurements, mathematically drying the air stream, background baselining for each of the two analyzers and correcting for room response using z‐transformation (Bartholomew et al., 1981). The theoretical basis for the equations is published by Lighton (2018). After data acquisition and processing, RMR is calculated using the Weir equation (Weir, 1949). Mean kcal/min was multiplied with 1440 to convert to kcal/day.

### 2.2 Calibration and equilibration of the instruments

Zero calibration of the gas analyzers of the GA3m2/FG‐250 was performed by first infusing nitrogen gas for 25 min and adjusting any CO_2_, O_2_, and WVP readings to zero. Secondly, a certified span gas with a CO_2_ concentration of 1.04% was infused for 5 min, and in the case of any deviation from the known concentrations of CO_2_, the respective sensors were adjusted to 1.04%. Finally, an automated equilibration procedure was performed to zero the WVP sensors in a process where incurrent air is passed through the desiccant canister. The span for WVP is calculated as the amount of WVP that would be present if the O_2_ readings were 20.94%, using the current WVP readings when the instruments are sampling incurrent air. Additional technical details can be found in (Lighton, 2018; Rising et al., 2015, 2017). A column desiccant (Drierite, Fisher Scientific) canister was used to chemically dry the air stream in order to zero the WVP sensors during calibration. Calibration and equilibration were performed prior to each day of testing.

### 2.3 Quality control and technical validation

Ten one‐h propane combustion tests using instrument‐grade propane (99.2% purity) (Airgas Healthcare) were performed in accordance with methods outlined in (Rising et al., 2017). Briefly, propane consumed (g) was measured by a calibrated scale (Sartorius Lab Instruments GmBH & Co), and the weight of the propane was noted at the start and end of the 1‐h measurement for the calculation of the propane burn rate (g/min).

### 2.4 Study participants for reproducibility/biological validation

For the reproducibility/biological validation study, students and staff at the Department of Nutrition, University of Oslo were recruited for two consecutive measurements separated by 24‐h between March and June in 2022. In total, 20 participants (6 men and 14 women) were included. One participant was excluded from the analysis because of illness at measurement Day 2, bringing the final number of participants to 19 (6 men and 13 women) (Table 2). Exclusion criteria were body mass index <18 kg/m^2^, medications that affect energy metabolism (e.g., Levaxin), smoking, chronic disease with known effects on energy expenditure, pregnancy, lactation, unstable weight the past 3 months (± 5%) and self‐reported claustrophobia. Participants gave written informed consents, and a letter of exempt was provided from the Regional Ethical Committee, as the project was considered methodological and not health research (Reference number: 368847). The project was approved by the Data Protection Services (SIKT) (Reference number: 359371).

### 2.5 Study protocol

**FIGURE 1:**
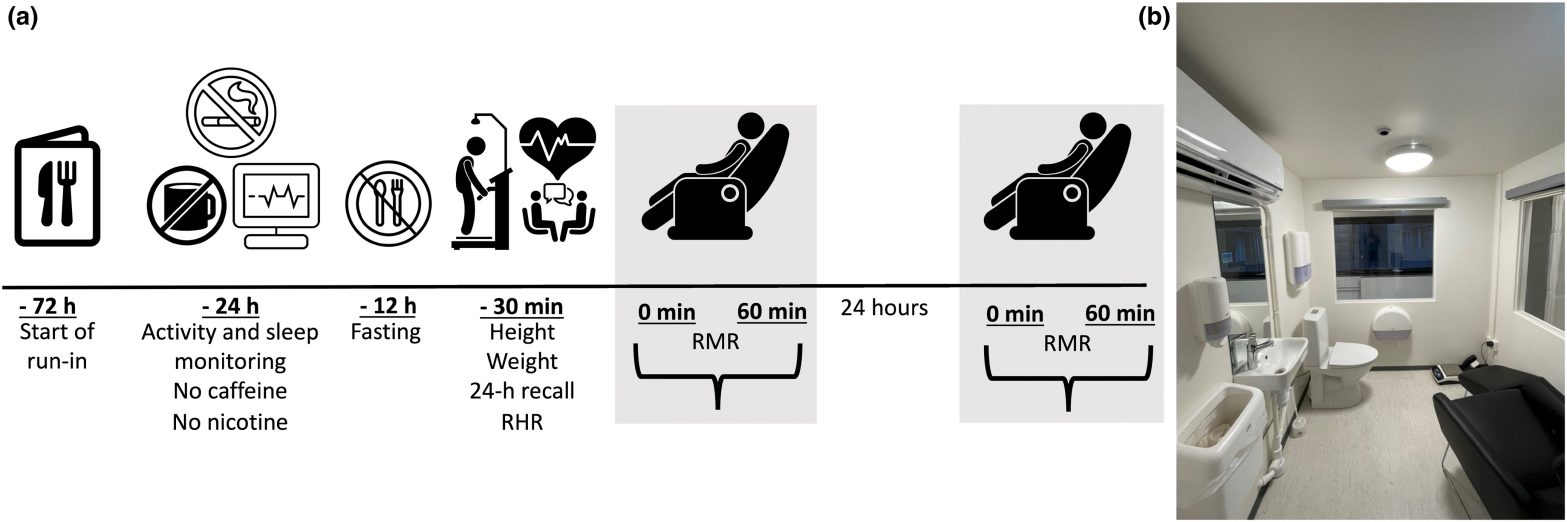
(a) Overview of the study design and (b) picture of the RMR‐WRIC at the Department of Nutrition, University of Oslo.

An overview of the study protocol can be seen in Figure 1a. Three days (−72 h) prior to each measurement, participants were advised to follow a diet in line with the food‐based dietary guidelines from the Norwegian Directorate of Health (Helsedirektoratet, 2015). Guidelines included eating the following:

1. Three to five meals per day
2. Five portions of fruits, berries and vegetables per day
3. Four portions of whole‐grain products per day
4. Three portions of low‐fat dairy per day
5. Meat‐, fish‐, or vegetarian‐based dinner
6. Potatoes, rice, or pasta for dinner, in the amounts needed to feel full
7. Use vegetable oils instead of butter for cooking
8. Avoid sugar‐sweetened beverages
9. Avoid alcohol

One day (−24 h) prior to each measurement, participants were asked to refrain from caffeine intake (coffee, tea, energy drinks, soda drinks) and vigorous exercise such as going to the gym or for a run. The participants were required to fast for 12 h and advised to sleep for 6 to 8 h prior to the measurement. Protocol adherence was checked by (1) a 24‐h dietary recall at the first measurement day, and (2) activity and sleep monitoring the final 24 h before measurements using SenseWear Armband Mini (BodyMedia), which has been validated for measuring physical activity and sleep in several populations (Berntsen et al., 2010; Cereda et al., 2007; MacKey et al., 2011; Reeve et al., 2014). Physical activity and sleep monitoring, and resting heart rate measurements were taken prior to each of the two measurements. The subjects were asked to maintain their self‐reported diet on the day before the second measurement. Significant protocol deviations that warranted sensitivity analyses were any consumption of caffeinated drinks, > 10 min of vigorous physical activity and non‐fasting measurements.

On the measurement days, height and weight measurements using a wireless scale (Seca 285 (Birmingham, UK)), and 24‐h recall interviews were performed immediately on arrival on Day 1. Brand names and recipe ingredients were registered. The food items were quantified by household measures and a booklet containing photographs of foods in different portion sizes (Norkost, 2012). Intake of foods were recorded as consumed and coded in the “Food composition database and food and nutrient calculation system (KBS),” at the department of Nutrition, University of Oslo. Participants rested for 30 min, and heart rate was recorded using a Polar wrist watch coupled to a heart rate monitor (Polar A300, Device ID 9F7511A, Firmware 1.2.135, HW model 00753320.07) before entering the WRIC. Sixty‐minute measurements started 5 min after closing the chamber door. The measurement was repeated after 24 h to minimize the influence of weight fluctuations and the menstrual cycle (Bisdee et al., 1989; Henry et al., 2003).

### 2.6 Statistical analysis

Distribution of continuous variables were checked by visual inspection of histograms and QQ‐plots. For the technical validation, expected vs. measured VO_2_, VCO_2_, energy expenditure, and RQ were compared using independent t‐tests. For reproducibility, the primary outcome variables were VO_2_, VCO_2_, RMR, and RQ. Repeated measures were compared using paired t‐tests. Correlational analyses for Day 1 and Day 2 were performed using Pearson correlation. Measures of accuracy and precision included crude relative mean differences between the two measurements and the coefficient of variation (CV, standard deviation [SD]/mean×100) for each subject. The intraclass correlation coefficient (ICC) was derived from linear mixed regression models with the outcome variable as the dependent variable, no independent variable, and a random term for subject ID. Pearson correlation coefficients and ICCs >0.9 were defined as strong or excellent based on terminology used in a previous report (Allerton et al., 2021). Bland–Altman plots were made to visualize limits of agreement (mean difference ± 1.96 SD), and presence of outliers in the data. To explore proportional bias, linear regression models were constructed where the difference between Days 1 and 2 for each outcome was regressed on the mean value of each parameter for each subject. A *p*‐value <0.05 was considered significant. All statistical analyses were conducted in R version 4.2.1. R packages used for data wrangling, plotting and statistical analysis included the *tidyverse* package compilation, *data. table, patchwork*, and *lmer*.

In the biological reliability study, subjects with poor adherence to the recommendations of caffeine intake and being vigorously physical active the day prior to the measurement were kept in the full analyses to judge the effect of varying protocol adherence on the measurements. Sensitivity analyses excluding these subjects were performed in order to assess whether protocol deviance affected the main results.

## 3 RESULTS

### 3.1 Technical validation

For the propane combustion tests (*n* = 10), the mean (SD) amount of propane burned was 13.79 ± 2.16 g with an average burn rate of 0.2279 ± 0.037 g/min. Details from the tests are given in Table 1. Briefly, the relative differences between measured and expected values ranged from −0.55 (1.11) % for VO_2_ to 1.32 (1.07) % for energy expenditure, whereas the CVs ranged from 0.67% for VO_2_ to 1.00% for energy expenditure. There was a near‐perfect correlation between expected and measured values (*r* > 0.99).

**TABLE 1:** Results from the technical validation (*n* = 10)^a^.

	Measured^b^	Expected^b^	Δ (%)^b^ ^,^ ^c^	CV (%)^d^	*r* ^e^	*p* ^g^
VO_2_ (l/g propane)	1.69 ± 0.05	1.68 ± 0.43	−0.55 ± 1.11	0.67	0.998	0.65
VCO_2_ (l/g propane)	1.00 ± 0.03	1.01 ± 0.03	0.92 ± 0.93	0.82	0.998	0.47
RQ	0.60 ± 0.01	0.60 ± 0.00	0.53 ± 1.10	0.67	NA^f^	0.16
EE, kcal/g propane	7.77 ± 0.22	7.88 ± 0.20	1.32 ± 1.07	1.00	0.998	0.29

Abbreviations: CV, coefficient of variation; EE, energy expenditure; RQ, respiratory quotient.

^**a**^ Based on 10 60‐min propane burns. All values except RQ have been normalized against propane burned.

^b^ Values are mean (standard deviation).

^c^ Deltas are measured‐expected.

^d^ CVs are standard deviation_measured + expected_/mean_measured + expected_ ×100.

^e^ Pearson's correlation coefficient between measured and expected values.

^f^ The standard deviation for RQ was zero (it is always 0.6 during propane combustion) and a coefficient could not be computed.

^g^ *p*‐values are derived from independent t‐tests comparing measured vs. expected values.

### 3.2 Baseline characteristics and protocol adherence

The 19 participants (*n* = 6 males, *n* = 13 females) were on average 39.6 ± 15 years old with a BMI of 23.5 ± 2.7 kg/m^2^. In the 24 h before each visit, the subjects spent a median (min, max) of 1 (0, 19) minute doing vigorous physical activity (Table 2). Median (min, max) sleep were 6.99 (6.00, 8.00) hours.

**TABLE 2:** Baseline and run‐in characteristics of the participants.

*n* = 19		
Males, *n* (%)	6 (31.6)	–

	Mean ± SD	Median (min, max)
Age, y	39.6 ± 15.3	32 (24, 75)
Anthropometric measurements		
Body weight, kg	69.1 ± 13.6	67.3 (54.7, 101)
Body mass index, kg/m^2^	23.5 ± 2.65	22.9 (19.5, 30.3)
Resting heart rate, bpm	60.9 ± 8.38	59 (53, 84)
Physical activity^a^		
Moderate physical activity^b^, min/day	138 ± 57.3	131 (51.0, 277)
Vigorous physical activity^c^, min/day	2.90 ± 4.70	1 (0,19)
Moderate‐to‐vigorous physical activity, min/day	141 ± 60.0	132 (51.0, 288)
Sleep, hours/day	7.01 ± 0.62	6.99 (6.00, 8.00)
Steps, *n*/day	9369 ± 4264	9043 (4268, 21,250)
Total energy expenditure from SWA, kcal/d	2513 ± 562	2371 (1882, 4337)
Activity energy expenditure from SWA, kcal/d	1116 ± 416	1067 (618, 2501)
Dietary intake		
Energy intake, kcal/d	2156 ± 601	2102 (1076, 3267)
Protein, E%	18.2 ± 3.8	17.3 (11.8, 27.7)
Carbohydrate, E%	45.5 ± 5.7	45.5 (30.4, 56.9)
Fat, E%	33.5 ± 7.14	32.6 (23.2, 55.8)
Fruits, berries and vegetables^d^, g/d	529 ± 187	515 (218, 876)
Whole grains^e^, g/d	78 ± 41	77.3 (7, 164)
Fish (total), g/d	56 ± 70	0.0 (0, 200)
Meat (total red and white meat), g/d	39 ± 54	15.0 (0, 174)
Dairy products^f^, g/d	384 ± 224	326.0 (38, 920)
Potatoes, g/d	31 ± 79	0.0 (0, 300)
Legumes, g/d	16 ± 46	0 (0, 180)
Sugary foods^g^, g/d	38 ± 36	32 (0, 127)
Margarine, butter, oils, g/d	26 (0, 107)	18 (0, 106)
Caffeinated drinks, *n*	2 ± 1	2 (1, 4)

Abbreviation: SWA, SenseWear Armband Mini.

^a^ *n* = 18.

^b^ Moderate intensity defined as 3–6 METS.

^c^ Vigorous intensity defined as >6 METS.

^d^ Include max one glass (200 grams) of juice as one portion of fruit (100 grams), not jam.

^e^ Intake of whole grains was calculated using a whole‐grain factor (with the assumption that bread contains 60% flour and boiled rice/pasta contains 30% cereal): Bread with 0%–25% wholemeal flour: (60*0)/10,000 = 0; Bread with 25%–50% wholemeal flour: (60*25)/10,000 = 0.15; Bread with 50%–75% wholemeal flour: (60*50)/10,000 = 0.30; Bread with 75%–100% wholemeal flour: (60*75)/10,000 = 0.45; Whole‐grain crisp bread = 1; Sweetened cereals = 0.25; Unsweetened cereals = 0.75; Brown rice = 0.30; Whole‐grain pasta = 0.30.

^f^ Includes milk, yoghurt, cheese, other dairy products.

^g^ Includes cakes, dessert, ice‐cream, chocolate and candy.

Total energy intake during the run‐in period was on median (min, max) 2102 (1076, 3267) kcal/d. Percent of energy from protein intake was 17 (12, 28), carbohydrate intake was 46 (30, 57), and fat intake was 33 (23, 56).

Of the 19 participants, one reported vigorous physical activity (biking to work) whereas five consumed caffeinated beverages −24 h before measurements. These were kept in the final analyses but excluded in sensitivity analyses reported under Sensitivity analyses and sources of RQ variability.

### 3.3 Biological reliability

Mean values, relative differences, deltas, CVs, ICCs, and correlation coefficients for each measurement day are shown in Table 3. VO_2_, VCO_2_, and RMR showed excellent reliability as assessed by ICC (94%–96%), with CVs at 2.89% for VO_2_, 2.67% for VCO_2_, and 2.68% for RMR. There were no significant differences between Day 1 and Day 2 for VO_2_, VCO_2_, and RMR. For RQ, ICC was lower at 74% with a CV of 1.95%, and there was a significant difference between Day 1 and Day 2 (mean difference = 0.02, *p* = 0.004).

**TABLE 3:** Accuracy and reliability of repeated measurements (*n* = 19).

	Day 1 mean ± SD	Day 2 mean ± SD	Δ (%)^a^	CV (%)^b^	ICC (%)^c^	*r* ^d^	*p* ^e^
VO_2_, L/day	355 ± 57.0	349 ± 58.4	−1.64 ± 4.77	2.89%	94%	0.95	0.17
VCO_2_, L/day	281 ± 45.5	282 ± 45.4	0.43 ± 4.21	2.67%	96%	0.96	0.77
RQ	0.79 ± 0.03	0.81 ± 0.04	2.17 ± 2.85	1.95%	74%	0.86	0.004
RMR, kcal/day	1710 ± 274	1690 ± 278	−1.26 ± 4.54	2.68%	95%	0.95	0.26

Abbreviations: CV, coefficient of variation; RMR, resting metabolic rate; RQ, respiratory quotient.

^a^ Deltas are Day 2—Day 1.

^b^ CVs are standard deviation_day 1 + day 2_/mean_day 1 + day 2_ × 100.

^c^ ICCs are derived from linear mixed model regression with the measured parameter as the outcome and a random term for subject ID.

^d^ Pearson's correlation coefficient between measured and parameters at Day 1 and Day 2.

^e^ *p*‐values are derived from paired *t*‐tests comparing values between Day 1 and Day 2.

**FIGURE 2:**
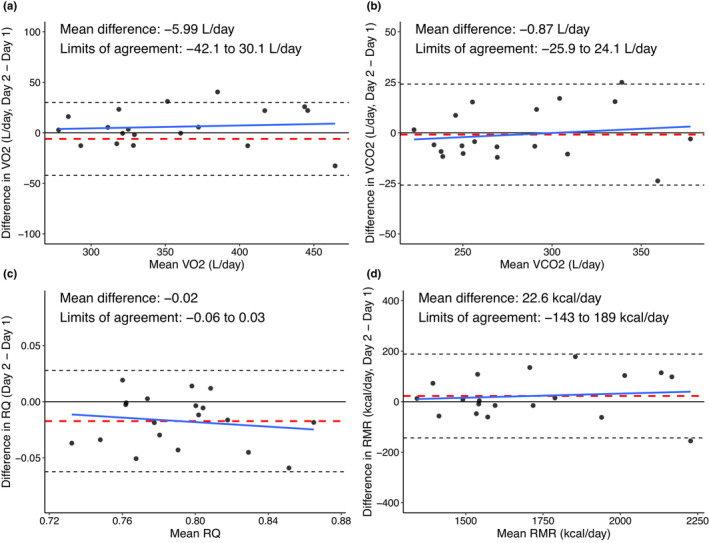
Bland–Altman plots with zero (black solid line), mean difference (red dashed line), limits of agreement (black dashed line) and regression line for proportional bias (blue solid line) for (a) VO_2_, (b) VCO_2_, (c) RQ, and (d) RMR. Abbreviations: RMR, resting metabolic rate; RQ, respiratory quotient.

The Bland–Altman plots in Figure 2 illustrate the mean difference, 95% limits of agreement and the regression line for proportional bias. For VO_2_, the limits of agreement ranged from −42.1 to 30.1 L/day; for VCO_2_ from −25.9 to 24.1 L/day; for RQ from −0.06 to 0.03; and for RMR from −143 to 189. There was no evidence of proportional bias indicating that there were no significant changes in the individual differences with higher values of VO_2_, VCO_2_, RQ, or RMR (all *p* > 0.90).

### 3.4 Sensitivity analyses and sources of RQ variability

With the exception of a decrease in the ICC for RQ from 74 to 69%, all parameters were generally similar in sensitivity analyses excluding non‐compliant subjects (Table 4).

**TABLE 4:** Accuracy and reliability of repeated measurements in sensitivity analyses excluding non‐compliant subjects (*n* = 14).

	Day 1 mean ± SD	Day 2 mean ± SD	Δ (%)^a^	CV (%)^b^	ICC (%)^c^	*r* ^d^	*p* ^e^
VO_2_, L/day	356 ± 57.5	349 ± 59.3	−1.87 ± 4.96	2.91%	94%	0.94	0.21
VCO_2_, L/day	280 ± 48.0	281 ± 48.3	0.58 ± 4.47	2.79%	96%	0.96	0.74
RQ	0.79 ± 0.03	0.81 ± 0.05	2.59 ± 3.03	2.22%	69%	0.85	0.008
RMR, kcal/day	1710 ± 278	1690 ± 284	−1.42 ± 4.73	2.70%	94%	0.95	0.31

Abbreviations: CV, coefficient of variation; RMR, resting metabolic rate; RQ, respiratory quotient.

^a^ Deltas are Day 2—Day 1.

^b^ CVs are standard deviation_day 1 + day 2_/mean_day 1 + day 2_ × 100.

^c^ ICCs are derived from linear mixed model regression with the measured parameter as the outcome and a random term for subject ID.

^d^ Pearson's correlation coefficient between measured and parameters at Day 1 and Day 2.

^e^ *p*‐values are derived from paired t‐tests comparing values between Day 1 and Day 2.

We and others (Allerton et al., 2021; Delsoglio et al., 2020) have observed less reliable measures of RQ following repeated measures using indirect calorimetry, possibly by accumulating random errors in VO_2_ and VCO_2_ measurements. Therefore, we explored whether the day‐to‐day variability in VO_2_ and VCO_2_ could predict the variability in RQ. There was no correlation between VCO_2_ residuals and RQ residuals (data not shown). However, there was a significant, inverse association between VO_2_ residuals and RQ residuals (Pearson's *r* = −0.52, *p* < 0.001).

## 4 DISCUSSION

The aim of this study was to evaluate a 7500 L WRIC in estimating VO_2_, VCO_2_, RMR, and RQ using propane combustion and repeated measurements within subjects, to allow for short‐term measurements of RMR which is less labor‐intensive than full 24‐h measurements. Overall, measured vs. expected values for the propane combustion tests were satisfactory, and VO_2_, VCO_2_, and RMR showed excellent test–retest reliability for the 19 subjects with ICCs ranging from 94 to 96%. For RQ, ICC was modest at 74% with a CV of 1.95%. These estimates were robust to protocol deviations in some participants.

For the technical validation, our results are comparable to a previous study (Rising et al., 2015) and were within ± 1.5% of expected values calculated from propane stoichiometry for all measures. For reliability, we observed slightly higher ICCs and lower CVs for RQ and RMR compared to repeated measurements taken in a 24‐h WRIC (Allerton et al., 2021). Although this observation may be due to differences in protocol, sample size, and instrumentation, smaller WRICs have shorter response times and require smaller correction factors for room volume during short‐term measurements, possibly increasing precision (Chen, Smith, et al., 2020). Related to this, a study comparing 40‐min measurements of RMR in one large (26,000 L) and one small (5500 L) WRIC observed a relative difference of ~4% between the two measurements (Chen, Scott, et al., 2020), possibly due to the difference in response time.

In contrast to VO_2_, VCO_2_, and RMR, repeated measures of RQ showed lower test–retest reliability and weaker correlation coefficients between measurements. The reason for this is uncertain, although some explanations have been offered by other authors. Desoglio et al. discuss that the lower reliability between measurements in RQ is due to the fact that random errors in between‐day measurements of VO_2_ and VCO_2_ accumulate and leads to inaccurate RQ measurements (Delsoglio et al., 2020) especially over short observation periods (Chen, Smith, et al., 2020). In addition, RQ is a ratio with a physiological range of 0.7 to 1.2 and small deviations in VO_2_ and VCO_2_ can therefore produce substantial noise. Indeed, longer measurements tend to produce higher ICCs for 24‐h RQ (Allerton et al., 2021; Dörner et al., 2022) whereas basal, sleeping, and resting RQ appear less reliable (Allerton et al., 2021). We observed a significant inverse correlation between scaled VO_2_ and scaled RQ residuals, indicating that as the variability in VO_2_ increases, so does the variability in RQ, whereas this was not observed for VCO_2_. As opposed to CO_2_ analyzers, the O_2_ analyzers are susceptible to any remaining water vapor in the sample gases after WVP dilution correction (Lighton, 2018). Thus, day‐to‐day variation in chamber humidity can produce small errors in the estimation of VO_2_, which may in turn compound with further calculations and impact the variability of RQ. The extension of this effect of VO_2_ variability on RQ is unreliable estimates of macronutrient oxidation. It has been shown that even small errors in VO_2_ can produce large errors in estimates of carbohydrate and fat oxidation (Livesey & Elia, 1988), which therefore represents a limitation of the current approach.

Although we show excellent reliability for components of energy expenditure, our study has some limitations. While we attempted to control dietary intake by giving food‐based advice, a greater degree of control could have been achieved if meals for the run‐in period had been prepared and tailored to the requirement of each subject. Although the subjects were asked to maintain their self‐reported diet, an additional 24‐h recall interview might have been performed to enforce greater control of the dietary data. Adherence to the dietary advice was acceptable during the run‐in, except for five participants who consumed caffeinated drinks and one who performed vigorous physical activity for 19 min the day before measurement in the WRIC facility. However, sensitivity analyses revealed no influence of protocol deviations on the measured parameters. An additional measurement day and measurements under different conditions (e.g., thermic effect of food) would also have been desirable to increase accuracy, precision and usability, and to be in line with previous validation studies (Allerton et al., 2021; Dörner et al., 2022; Schoffelen & Westerterp, 2008), but was not possible due to resource constraints.

## 5 CONCLUSION

In conclusion, we have demonstrated that a 7500 L WRIC measures VO_2_, VCO_2_, and RMR with excellent reliability, whereas RQ was measured with acceptable reliability. These findings are of crucial importance for future research and clinical applications with RMR as the outcome.

## AUTHOR CONTRIBUTIONS

TO conceived the idea for the study; HBH, CH, RD, FH, SMU, and TO designed the study; HBH, CH, ARSS, DTA, EMSA, and TO collected data; RR customized the macro files used to estimate metabolic rates; ABW and RB made intellectual contributions; HBH and TO drafted the paper; All authors read, revised, and approved the final version of the paper.

## FUNDING INFORMATION

This project received support from Direktør Throne Holsts Fond For Ernæringsforskning and the University of Oslo.

## CONFLICT OF INTEREST STATEMENT

The authors declare no conflicts of interest.
